# Wie beeinflusst die präoperative Wartezeit die Krankenhaussterblichkeit und Komplikationsrate bei geriatrischen Patienten mit medialer Schenkelhalsfraktur?

**DOI:** 10.1007/s00113-025-01575-w

**Published:** 2025-05-05

**Authors:** Annette Keß, Johanna Krauße, Philipp Pieroh, Christian Kleber, Johannes Fakler, Georg Osterhoff

**Affiliations:** 1 Klinik für Orthopädie, Unfallchirurgie und Plastische Chirurgie, Universitätsklinikum AöR Leipzig, Liebigstraße 20, 04103 Leipzig, Deutschland; 2 Klinik für Orthopädie und Unfallchirurgie, Klinikum Passau, Passau, Deutschland

**Keywords:** Proximale Femurfraktur, Mortalität, Hemiarthroplastik, Unfallchirurgie, Geriatrie, Proximal femoral neck fractures, Mortality, Hemiarthroplasty, Trauma surgery, Geriatrics

## Abstract

**Fragestellung:**

Die aktuelle Richtlinie des Gemeinsamen Bundesausschusses fordert eine frühestmögliche operative Versorgung hüftgelenknaher Femurfrakturen innerhalb von 24 h nach Aufnahme zur Senkung der Komplikationsrate und Mortalität.

**Ziel der Arbeit:**

Ziel war es, Krankenhaussterblichkeit sowie Komplikationsrate und -arten in Bezug auf die präoperative Wartezeit zu analysieren.

**Methodik:**

Zwischen 2010 und 2020 wurden 575 Patienten mit Duokopfprothese nach Schenkelhalsfraktur retrospektiv hinsichtlich Krankenhausmortalität und Komplikationen untersucht. Ausgeschlossen wurden pathologische Frakturen, Frakturen älter als 4 Wochen sowie osteosynthetisch versorgte Patienten. Erfasst wurden patientenspezifische Daten, Krankenhaussterblichkeit sowie Komplikationsrate und -arten.

**Ergebnisse:**

Die präoperative Wartezeit sank im Median von 38 h (2010) auf 19 h (2020). Patienten, die innerhalb von 24 h operiert wurden, erhielten die OP im Median nach 14,2 h, im Vergleich zu 40,2 h bei späterer OP. Der ASA-Mittelwert lag bei 2,76; die > 24 h-Gruppe wies signifikant höhere ASA-Werte auf (*p* = 0,024). 12 (4,2 %) Patienten starben in der  24 h-Gruppe (*p* = 0,035). Die Komplikationsrate lag bei 15 % (88 Patienten), ohne signifikanten Unterschied zwischen den Gruppen.

**Schlussfolgerungen:**

Die präoperative Wartezeit halbierte sich auf 19 h. Die Gruppe der Patienten mit OP innerhalb 24 h relativierte diesen Effekt jedoch; nach Adjustierung war die 24 h-Grenze kein unabhängiger Risikofaktor. Die Komplikationsraten unterschieden sich nicht signifikant.

**Graphic abstract:**

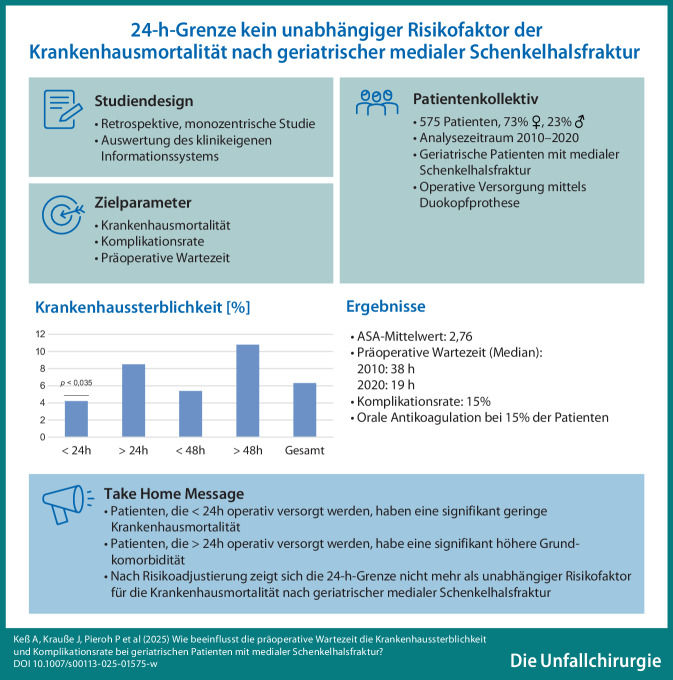

## Abstract (en)

**Background:**

The current guidelines of the German Federal Joint Committee on the treatment of proximal femoral fractures require that patients with a proximal femoral fracture receive surgical treatment as soon as possible and within 24 h of admission. This is intended to reduce perioperative complication rate and mortality.

**Objective:**

The aim of this study was to analyze the hospital mortality as well as complication rates and types in relation to the preoperative waiting time.

**Methodology:**

From 2010 to 2020, a total of 575 patients with femoral neck fractures treated with a bipolar prosthesis were retrospectively analyzed with respect to hospital mortality and the occurrence of complications. Patients with pathological fractures, femoral neck fractures more than 4 weeks old and those treated with osteosynthesis were excluded. Patient-specific data, hospital mortality and complication rates and types were recorded.

**Results:**

During the study period, the implementation of the given guidelines resulted in a reduction in the preoperative waiting time from a median of 38 h in 2010 to 19 h in 2020. Surgical treatment was performed on average after 14.2 h in patients who had surgery within 24 h after admission and on average after 40.2 h for those who had surgery after 24 h. The average American Society of Anesthesiologists (ASA) score for all 575 patients was 2.76. The group of patients who had surgery after more than 24 h had a significantly higher ASA classification (*p* = 0.024). A total of 12 (4.2%) patients in the group surgically treated within 24 h died, compared to 24 (8.5%) deaths in the group surgically treated after 24 h (*p* = 0.035). The complication rate for the entire cohort was 15% (88 patients). There was no difference in the occurrence of complications and the overall complication rate with respect to the timing of surgery.

**Conclusion:**

During the study period the preoperative waiting time was halved from 39h to 19 h. Patients who were surgically treated within 24 h had a significantly lower hospital mortality than those surgically treated after 24 h; however, the group surgically treated after 24 h simultaneously showed higher baseline comorbidities and in the adjusted analysis for age and ASA score, the 24‑h threshold no longer emerged as an independent risk factor for hospital mortality. Regarding complication rates, no significant differences were found between the groups based on the timing of surgery.

## Einleitung

Hüftgelenknahe Frakturen gehören mit rund 100.000 Fällen/Jahr zu den häufigsten osteoporosebedingten Frakturen in Deutschland und betreffen v. a. ältere und geriatrische Patienten [8] mit steigenden Inzidenzen bei steigendem Alter [23, 30].

Im Beschluss des Gemeinsamen Bundesausschusses über die Richtlinie zur Versorgung der hüftgelenknahen Femurfrakturen vom Dezember 2020 wurde eine Gewährleistung einer frühestmöglichen operativen Versorgung von Patienten mit einer hüftgelenknahen Femurfraktur innerhalb von 24 h nach Aufnahme oder nach Auftreten eines innerklinischen Sturzes festgelegt[3, 9]. Dies soll die perioperative Morbidität und Mortalität senken, wobei in der Literatur Zeiträume zwischen Aufnahme und operativer Versorgung von 24–48 h diskutiert werden [7, 14, 20, 25, 27, 32]. Kürzere Zeiträume gehen auch mit einem höheren perioperativen logistischen Aufwand einher.

Als operative Versorgungsstrategien für mediale Schenkelhalsfrakturen stehen neben der Osteosynthese mittels dynamischer Hüftschraube (DHS) der endoprothetische Ersatz mit einer Hüfttotalendoprothese (HTEP) bzw. Duokopfprothese im Vordergrund. Besonders bei geriatrischen Patienten mit bereits präoperativer Multimorbidität und eingeschränkter Mobilität erfolgt die Versorgung überwiegend mittels Duokopfprothese [5].

Aktuell liegt die Einjahresmortalität nach operativer Versorgung einer hüftgelenknahen Fraktur bei rund 19–25 % [2, 10, 11, 13, 17], während die Krankhaussterblichkeit zwischen 2 und 7,6 % liegt [1, 5, 10, 12, 24]. Die häufigsten chirurgischen Komplikationen sind die Wundheilungsstörung, Frühinfektion, ein postoperatives Serom/Hämatom und periprothetische Frakturen, welche erheblichen Einfluss auf die Lebensqualität haben. Chirurgische Komplikationen treten im Umfang von 3,6 % [5] bis 22 % [19, 20] auf.

Ziel dieser Analyse war es die, die Krankenhaussterblichkeit sowie Komplikationsrate und -arten ,bezogen auf die präoperative Wartezeit, zu analysieren.

## Methodik

### Studiendesign

Die Studie basiert auf der Weiterverwendung gesundheitsbezogener Daten eigenbehandelter Patienten nach § 29 des Sächsischen Krankenhausgesetzes; eine gesonderte Aufklärung erfolgte nicht. Die Studie wurde von der lokalen Ethikkommission bewertet (Aktenzeichen 323/23-ek).

In dieser retrospektiven Studie wurden die Daten von 575 Patienten analysiert, welche im Zeitraum von 2010 bis 2020 nach medialer Schenkelhalsfraktur mittels Duokopfprothese an unserem universitären Traumazentrum operativ versorgt wurden. Eingeschlossen wurden alle Patienten, die zum Operationszeitpunkt 65 Jahre oder älter waren. Ausgeschlossen wurden Patienten mit pertrochantärer Femurfraktur, pathologischen Frakturen, Frakturalter älter als 4 Wochen sowie vorangegangener Osteosynthese oder Implantation einer Totalendoprothese.

Die Datenerhebung erfolgte im klinikinternen Informationssystem unter Auswertung der schriftlichen Dokumentation sowie der Laborwerte und Röntgenbilder. Ausgewertet wurden patientenspezifische Daten, Krankenhaussterblichkeit, Rate chirurgischer Komplikationen und -arten und Revisionsoperationen sowie Zeit und Dauer der initialen Operation, verwendete Implantate und Zement, Zugangsweg; außerdem wurden intraoperative Komplikationen dokumentiert.

### Statistik

Alle Daten wurden in einer Excel-Datenbank (Microsoft Corp., Washington, DC, USA) erfasst und zur statistischen Analyse in SPSS 27.0 (SPSS Inc., Chicago, IL, USA) exportiert. Sofern nicht anders angegeben, wurden die Daten als Mittelwert mit Standardabweichung zusammengefasst.

Primärer Endpunkt war die Krankenhausmortalität, also das Versterben während des stationären Aufenthalts. Es wurde eine Regressionsanalyse durchgeführt, um die „Zeit bis zur Operation“ als potenziellen unabhängigen Risikofaktor für die Krankenhausmortalität zu bewerten. Um für die mit der Krankenhaussterblichkeit assoziierten Störfaktoren Alter und Komorbidität (ASA-Score) zu adjustieren, erfolgte eine binär-logistische Regressionsanalyse, und es wurden „odds ratios“ (OR) mit 95 %-Konfidenzintervallen (95 %-KI) berechnet.

Die erhobenen Datensätze können auf begründete Anfrage in anonymisierter Form beim korrespondierenden Autor angefordert werden. Die Daten befinden sich auf einem Datenspeicher am Universitätsklinikum Leipzig.

## Ergebnisse

**Abb. 1:**
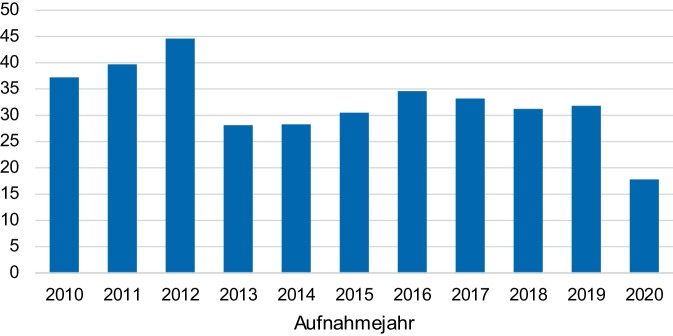
Präoperative Wartezeit

Das Patientenkollektiv war zu 73 % weiblich und zu 27 % männlich. Bei 50,3 % der Patienten erfolgte die Operation innerhalb von 24 h, bei 85,2 % innerhalb von 48 h nach Aufnahme. Die Fraktur befand sich zu 52 % auf der linken Seite, zu 48 % rechts und zu 0,2 % beidseits. Zu 8,7 % handelte es sich um nichtdislozierte Frakturen der Typen I und II in der Garden-Klassifikation und zu 91,2 % um dislozierte Frakturen der Typen III und IV nach Garden (Tab. 1). Im Analysezeitraum von 2010 bis 2020 zeigte sich unter Umsetzung der gegebenen Richtlinien eine Reduktion der präoperativen Wartezeit von im Median 38 h im Jahr 2010 auf 19 h in 2020 (Abb. 1). Hierbei erfolgte die Operation im Median nach 14,2 h bei den innerhalb von 24 h operierten Patienten und nach 40,2 h für die Patienten, die erst nach 24 h operativ versorgt wurden.

**Tab. 1:** Epidemiologie des Patientenkollektivs

Parameter	Gesamtkollektiv (*n* [%])	Operation ≤ 24 h (*n* [%])	Operation ≤ 48 h (*n* [%])	*p*-Wert
Anzahl, Geschlecht w:m	422 [26,6]:153 [73,2]	221 [76,5]:68 [23,5]	201 [70,3]:85 [29,7]	0,093
Alter (*MW* ± *SD*)	82,5 ± 8,6	82,5 ± 8,6	81,9 ± 8,8	0,531
*ASA-Klassifikation*				
1	6 [1,0]	4 [1,4]	2 [0,7]	0,024
2	142 [24,7]	84 [29,1]	58 [20,3]	
3	412 [71,7]	197 [68,2]	215 [75,2]	
4	15 [2,6]	4 [1,4]	11 [3,8]	
*Garden-Klassifikation*				
1	11 [1,9]	8 [2,8]	3 [1,1]	0,402
2	39 [6,8]	17 [5,9]	22 [7,7]	
3	328 [57,1]	166 [57,4]	162 [56,8]	
4	196 [34,1]	98 [33,9]	98 [34,3]	
Präoperative Wartezeit	31,8 h	14,3 h	49,6 h	–

*w* weiblich, *m* männlich, *MW* Mittelwert, *SD* Standardabweichung

Hinsichtlich der Komorbiditäten wiesen 28 % der Patienten einen Diabetes mellitus, 15 % eine Malignomerkrankung, 13 % neurologische Erkrankungen (u. a. Epilepsie), 28 % eine Demenz und 2 % relevante dermatologische Erkrankungen auf. Aktive Raucher waren 11 % der Patienten, 1 % gab an, regelmäßig Alkohol zu trinken, und 15 % der Patienten nahmen eine orale Antikoagulation ein (Tab. 2).

**Tab. 2:** Übersicht der Komorbiditäten

Parameter	Gesamtkollektiv (*n* [%])	Operation ≤ 24 h (*n* [%])	Operation ≤ 48 h (*n* [%])
Demenz	162 [28,2]	74 [12,9]	137 [23,8]
Diabetes mellitus	161 [28,0]	72 [12,5]	133 [23,1]
Kardiale Vorerkrankungen	461 [80,2]	225 [39,1]	399 [69,4]
Orale Antikoagulation	91 [15,8]	32 [5,6]	70 [12,2]
Malignomerkrankung	6 [1,0]	3 [0,5]	6 [1,0]
Immunsuppression	14 [2,4]	10 [1,7]	13 [2,3]
Dermatologische Erkrankungen	10 [1,7]	7 [1,2]	9 [1,6]
Neurologische Erkrankungen	71 [14,1]	33 [5,7]	61 [10,6]
Aktive Raucher	62 [10,8]	27 [4,7]	50 [8,7]
Substanzabusus	5 [0,9]	1 [0,2]	5 [0,9]

**Abb. 2:**
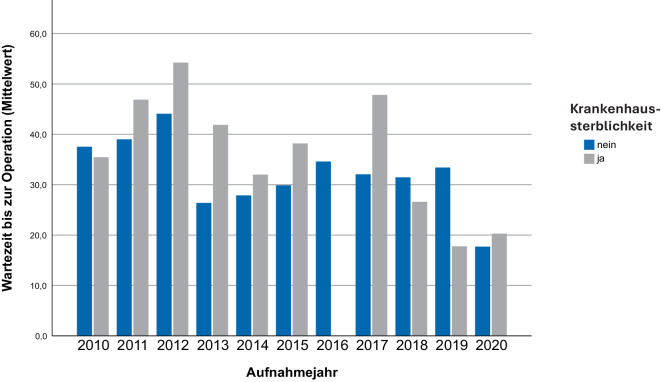
Krankenhaussterblichkeit, bezogen auf die mittlere präoperative Wartezeit je Aufnahmejahr

Der ASA-Mittelwert aller 575 operierten Fälle liegt bei 2,76. Bei genauer Aufschlüsselung weist die Gruppe der in > 24 h operativ versorgten Patienten eine signifikant höhere ASA-Klassifikation (ASA-Mittelwert 2,82) auf als die Gruppe der in < 24 h operierten Patienten (ASA-Mittelwert 2,7, *p* = 0,024). Auch hinsichtlich der Krankenhaussterblichkeit zeigen sich signifikante Unterschiede zwischen den Gruppen. Es verstarben 12 (4,2 %) Patienten, die innerhalb von 24 h versorgt wurden, während ihres primären Krankenhausaufenthalts, im Vergleich zu 24 (8,5 %) Verstorbenen in der Gruppe der nach 24 h operativ versorgten Patienten (*p* = 0,035) (Tab. 3; Abb. 2).

**Tab. 3:** Krankhaussterblichkeit in Abhängigkeit von der präoperativen Wartezeit

Präoperative Wartezeit	Krankenhaussterblichkeit (*n* [%])	*p*-Wert
Gesamt	36 [6,3]	–
< 24 h	12 [4,2]	–
> 24 h	24 [8,5]	0,035
< 48 h	26 [5,4]	–
> 48 h	10 [10,6]	0,026

Nach Adjustierung für die mit einer erhöhten Krankenhausmortalität assoziierten Risikofaktoren Alter (OR 1,05, 95 %-KI [1,02, 1,08]) und ASA-Score (OR 4,61, 95 %-KI [2,67, 7,96]) ergab sich für die Patienten, die innerhalb von 24 h operiert wurden, keine statistisch signifikant erniedrigte Krankenhausmortalität mehr (OR 0,77, 95 %-KI [0,52, 1,16]).

Im gesamten Kollektiv betrug die Rate an Komplikationen innerhalb von 3 Monaten postoperativ 15 % (88 Patienten). Dabei kam es in 7,7 % der Fälle (44 Patienten) zu einer Revisionsoperation, in jeweils 6,6 % der Fälle (38 Patienten) ereigneten sich eine intraoperative Fraktur oder ein Trochanter-major-Abriss. Frühinfektionen traten in 5,2 % der Fälle (30 Patienten) auf. Insgesamt 477 Fälle (83 %) verliefen ohne jegliche Komplikationen (Tab. 4). Bezüglich des Auftretens der Komplikationen und der Gesamtkomplikationsrate bestand kein Unterschied bezüglich des Versorgungszeitpunktes, außer für eine kleine Stichprobe von Trochanter-major-Abrissen/Frakturen, welche bei < 24 h Operierten signifikant häufiger auftraten (*p* = 0,048). Zusätzlich zeigte sich, dass oral antikoagulierte Patienten (Vitamin-K-Antagonisten und direkte orale Antikoagulanzien) signifikant seltener innerhalb 24 h operiert wurden als nicht oral antikoagulierte Patienten (20,6 % (59/286) der > 24 h Operierten vs. 11,1 % (32/289) < 24 h Operierten antikoaguliert; (*p* = 0,002).

**Tab. 4:** Postoperative Komplikationen

Komplikationen	Gesamtkollektiv [%]	Operation ≤ 24 h [%]	Operation ≤ 48 h [%]	*p*-Wert
Revisionsoperation	7,7	6,6	8,7	0,328
Serom	4,2	5,5	2,8	0,101
Wundheilungsstörung	20,5	14,3	31	0,077
Frühinfektion	5,2	4,8	5,6	0,680
Spätinfektion	0,6	0,4	0,4	0,998
Trochanter-major-Fraktur	6,6	8,7	4,5	0,481
Intraoperative Fissur	1	0,3	1,7	0,981
Luxationen	2,1	2,8	1,4	0,251
Periprothetische Fraktur	1	0,7	1,4	0,404

## Diskussion

Der Einfluss der präoperativen Wartezeit auf Mortalität und Komplikationsraten sowie der optimale operative Versorgungszeitraum nach einer Schenkelhalsfraktur wurden und werden immer wieder kontrovers diskutiert. Bereits im Zeitraum von 1993 bis 1997 konnte [28] eine Reduktion der Krankenhaussterblichkeit von 6,9 % auf 5,4 % bei sinkender präoperativer Wartezeit von 2,6 auf 2,1 Tage beobachtet werden. Auch unsere Studie zeigte sinkende mittlere präoperative Verweildauern von 38 auf 19 h (1,63 auf 0,80 Tage) von 2010 bis 2020. Gleichzeitig zeigte sich in unseren Daten eine signifikant steigende Krankenhausmortalität bei operativer Versorgung nach mehr als 24 h. In der Gruppe der nach 24 h Operierten fand sich in unserer Kohorte jedoch gleichzeitig eine höhere Grundkomorbidität, und in der für Alter und ASA-Score adjustierten Analyse bestätigte sich die 24 -h-Grenze nicht mehr als unabhängiger Risikofaktor für die Krankenhausmortalität. Dies wies auch eine kanadische Metaanalyse von 42.230 Patienten mit hüftgelenknaher Fraktur nach. Die Studie zeigte eine durchschnittliche 30-Tage-Mortalität von 7 %, wobei die innerhalb 24 h Operierten signifikant seltener verstarben als > 24 h Operierte (5,8 % vs. 6,5 %; [22]). Zudem zeigte sich in der Gruppe der > 24 h Operierten ein signifikanter Anstieg von Komplikationen wie Lungenarterienembolie, Myokardinfarkt und Pneumonie [22]. Für chirurgische Komplikationen ließ sich kein signifikanter Unterschied zwischen den früher und später als in 24 h operierten Patienten identifizieren [22]. Eine weitere Studie konnte ebenfalls nachweisen, dass die Rate von Frühkomplikationen und -revisionen sowie die Mortalität bei den Patienten, die nach 24 h operiert wurden, signifikant erhöht waren [20]. Dabei hatten die nach 24 h operierten Patienten eine um 31,9 % erhöhte Wahrscheinlichkeit zu versterben [20]. Müller-Mai et al. [20] beschreiben einen allgemeinen Rückgang der präoperativen Wartezeit im Verlauf der letzten Jahre. So wurden beispielsweise nur 8 % der Patienten der Studie im Zeitraum von 2007 bis 2008 erst nach 48 h operiert. Die mittlere präoperative Verweildauer der Studie lag bei 1,46 Tagen (35 h), und 77,1 % der Patienten wurden innerhalb 24 h operiert [20]. In unserer Studie wurden im Vergleich 50,3 % (*n* = 289) innerhalb 24 h und 14,8 % (*n* = 85) der Patienten nach mehr als 48 h operiert; die mediane präoperative Liegezeit betrug 24,1 h. Dabei muss bei Bewertung des Medianwerts die Halbierung der präoperativen Wartezeit innerhalb des 10-jährigen Analysezeitraums von 39 auf 19 h beachtet werden. Frühere Studien [28] aus dem Analysezeitraum 1993–1997 zeigten bereits eine Reduktion der Liegezeiten von 2,6 auf 2,1 Tage bei jedoch gehäufter operativer Versorgung nach 48 h. In einer weiteren deutschen Studie von 2004 bis 2006 wurden 17,2 % der Patienten mit medialer Schenkelhalsfraktur erst nach 48 h operativ versorgt [27]. Diese Gruppe wies signifikant mehr chirurgische und allgemeine perioperative Komplikationen auf (*p* < 0,001), ohne signifikanten Anstieg der Krankenhaussterblichkeit (OR 0,96; *p* = 0,302) [27]. Dem entgegengesetzt zeigten die dänischen Autoren Öztürk et al. [21] eine steigende 30-Tage-Mortalität bei Operationen nach > 24 h, besonders bei Patienten mit wenigen bis mittelschweren Komorbiditäten. Bei Patienten mit schweren Komorbiditäten hatte die verlängerte Wartezeit keinen steigernden Einfluss auf die 30-Tage-Mortalität. Kristiansson et al. [15] wiesen in ihrer Analyse von Hüftfrakturen in Schweden von 2007 bis 2016 ebenfalls eine signifikant höhere 30-Tage-Mortalität für Patienten mit einer präoperativen Wartezeit von mehr als 48 h (*p* = 0,0012) nach. Sie ermittelten ab dem Zeitpunkt von 36 h Wartezeit einen Trend zu erhöhter Sterblichkeit, der ab 39 h statistisch signifikant wird, und sehen eine maximale Wartezeit von 24 h präoperativ als kritisch. Eine systematische Metaanalyse von 31.242 Patienten fand nach Risikoadjustierung keine statistisch signifikanten Unterschiede hinsichtlich der Mortalität bei innerhalb 24 h vs. später operierten Patienten (RR 0,82, 95 %-KI 0,67–1,01) [13]. Eine signifikant erhöhte Mortalität und Komplikationsrate zeigten sich nur in der Gruppe der nach 48 h Operierten [13]. Beide Studien zeigten keine signifikanten Mortalitätsunterschiede in der Gruppe von innerhalb 24 h Operierten, sondern erst ab 48 h, wobei im Unterschied zu unserer Studie nicht die Krankenhausmortalität, sondern die 30-Tage-Mortalität als Referenzwert genutzt wurde. Dem widerspricht eine britische Studie [4], die im Zeitraum von 1989 bis 2013 lediglich eine signifikant niedrigere 30-Tage-Mortalität bei einer innerhalb von 12 h erfolgten Operation (*p* = 0,013) berichtete, während weitere Verzögerungen bis zur Operation keine signifikanten Unterschiede zeigten. Zusätzlich zeigte sich der ASA-Wert als stärkster Prädiktor der 30-Tage-Mortalität, wobei jeder ASA-Grad höher eine steigende Odds Ratio (OR) von 2,52 bedeutet (95 %-KI 2,01–3,04; *p* < 0,001) [4]. Als weitere signifikante Prädikatoren für eine erhöhte 30-Tage-Mortalität wurden steigendes Alter (*p* < 0,001), extrakapsuläre Frakturen (*p* = 0,019), männliches Geschlecht (*p* = 0,014) und schlechte präoperative Mobilität (*p* < 0,001) identifiziert. Der ASA-Wert zeigte sich auch in weiteren Studien als konstanter Prädikator für eine erhöhte Mortalität von Patienten mit Hüftfrakturen, sowie männliches Geschlecht und erhöhtes Alter als Prädiktoren für ein schlechtes Outcome [11, 17, 31]. In einer Metaanalyse [6] von 9 Studien zu präoperativen Wartezeiten, Komplikationen und Mortalität bei Hüftfrakturen zeigte sich, dass die präoperative Wartezeit von Patienten mit DOAK (neuen oralen Antikoagulanzien) signifikant verlängert war. Dieser Effekt der signifikant längeren präoperativen Wartezeit ist auch in unserer Studie zu sehen.

Patienten mit oraler Antikoagulation wurden im Studienkollektiv häufiger erst nach > 24 h operiert. Grund dafür war in der Regel das Abwarten einer präoperativen Normalisierung der Gerinnung. Die Notwendigkeit einer oralen Antikoagulation ist assoziiert mit thrombembolischen oder kardiovaskulären Erkrankungen und damit auch mit einer höheren Mortalität. Allerdings führen diese auch zu einer höheren Einstufung in der ASA-Klassifikation, sodass die in dieser Studie durchgeführte Adjustierung die statistische Verzerrung durch die ungleiche Verteilung innerhalb des Kollektivs ausreichend adressiert.

In 8 der Studien fand sich kein signifikanter Unterschied der postoperativen Mortalität der antikoagulierten Patienten. Die 30-Tage-Mortalität belief sich auf 4,9 % vs. 5,2 % in der DOAK- und der Kontrollgruppe [6]. Zusammenfassend zeigen die aufgeführten Studien [20–22, 26]), dass Patienten von einer zeitnahen operativen Versorgung innerhalb von 24 h (bzw. mindestens im Zeitfenster 12–48 h) [4, 18] profitieren und dadurch eine geringere Mortalität verzeichnen. ASA-Werte von 3 und höher scheinen ein valider Marker zur Identifizierung dieser Patienten zu sein [11, 17, 29, 31]. Unterschiedliche Endpunkte und Beobachtungszeiträume erschweren den Vergleich der Studien. Zudem erfolgte nicht in allen Studien eine Risikoadjustierung für Komorbiditäten, die eine Unterscheidung von komorbiditätenbedingter Mortalität und Mortalität aufgrund verlängerter präoperativer Wartezeit ermöglichen würde [13, 16, 18]. Hier bedarf es weiterer Analysen. Die in unserer Analyse untersuchte Krankenhausmortalität erfolgte nur über einen kurzen postoperativen Beobachtungszeitraum; eine längerfristige Nachbeobachtung über beispielsweise ein Jahr erfolgte nicht. Zudem konzentrierten wir uns in dieser Studie auf chirurgische Komplikationen, und es erfolgte keine gesonderte Auswertung nichtchirurgischer Komplikationen, wie z. B. Pneumonien und Thrombosen.

Aufgrund des retrospektiven Designs der Studie war zudem eine vollständige Erfassung der einzelnen Komorbiditäten nicht möglich, sodass als Surrogatparameter für die Komorbidität die ASA-Klassifikation, welche für alle Patienten vollständig dokumentiert war, gewählt wurde. Anhand der vorliegenden Daten war es daher nicht möglich zu detektieren, ob einzelne Komorbiditäten (z. B. kardiovaskuläre Erkrankungen) als unabhängiger Risikofaktor für die Mortalität wirkten. Hierfür sind prospektive Studien mit größerer Fallzahl notwendig.

## Schlussfolgerung

Bezüglich der präoperativen Wartezeit zeigt sich im Analysezeitraum seit regulatorischer Implementierung der 24-h-Regel eine Halbierung von vorher im Mittel 38 auf nun 19 h. Patienten, die innerhalb von 24 h operativ versorgt wurden, wiesen eine signifikant geringere Krankenhausmortalität auf als Patienten, die nach 24 h operiert wurden. In der Gruppe der nach 24 h operierten fand sich jedoch gleichzeitig eine höhere Grundkomorbidität, und in der für Alter und ASA-Score adjustierten Analyse bestätigte sich die 24 -h-Grenze nicht mehr als unabhängiger Risikofaktor für die Krankenhausmortalität. Hinsichtlich der Morbidität konnten wir keine signifikanten Unterschiede zwischen den Gruppen nachweisen. Hinsichtlich der Komplikationsraten konnten wir, bis auf eine kleine Subgruppe, keine signifikanten Unterschiede zwischen den Gruppen nachweisen.

## Fazit für die Klinik

- Patienten, die < 24 h operativ versorgt werden, haben eine signifikant geringe Krankenhausmortalität.
- Patienten, die > 24 h operativ versorgt werden, habe eine signifikant höhere Grundkomorbidität.
- Komorbiditäten haben deutlichen Einfluss auf die Krankenhausmortalität.
- Nach Risikoadjustierung zeigt sich die 24 -h-Grenze nicht mehr als unabhängiger Risikofaktor für die Krankenhausmortalität nach geriatrischer medialer Schenkelhalsfraktur.

## Data Availability

Die erhobenen Datensätze können auf begründete Anfrage in anonymisierter Form beim korrespondierenden Autor angefordert werden. Die Daten befinden sich auf einem Datenspeicher am Universitätsklinikum Leipzig.
